# Supplementary material for: Association between oral microbiome and seven types of cancers in East Asian population: a two-sample Mendelian randomization analysis
Source: Front Mol Biosci. 2023 Nov 21;10:1327893. doi: 10.3389/fmolb.2023.1327893 (PMC10702768; doi:10.3389/fmolb.2023.1327893)
Supplement: Supplementary file 1 [file DataSheet1.ZIP › Supplementary Materials/MR plots for tongue/tongue═╝/Pancreatic cancer/╥╚╧┘░⌐_╔╕╤í╜ß╣√/pheno.824_to_pancreatic cancer_forest.pdf]

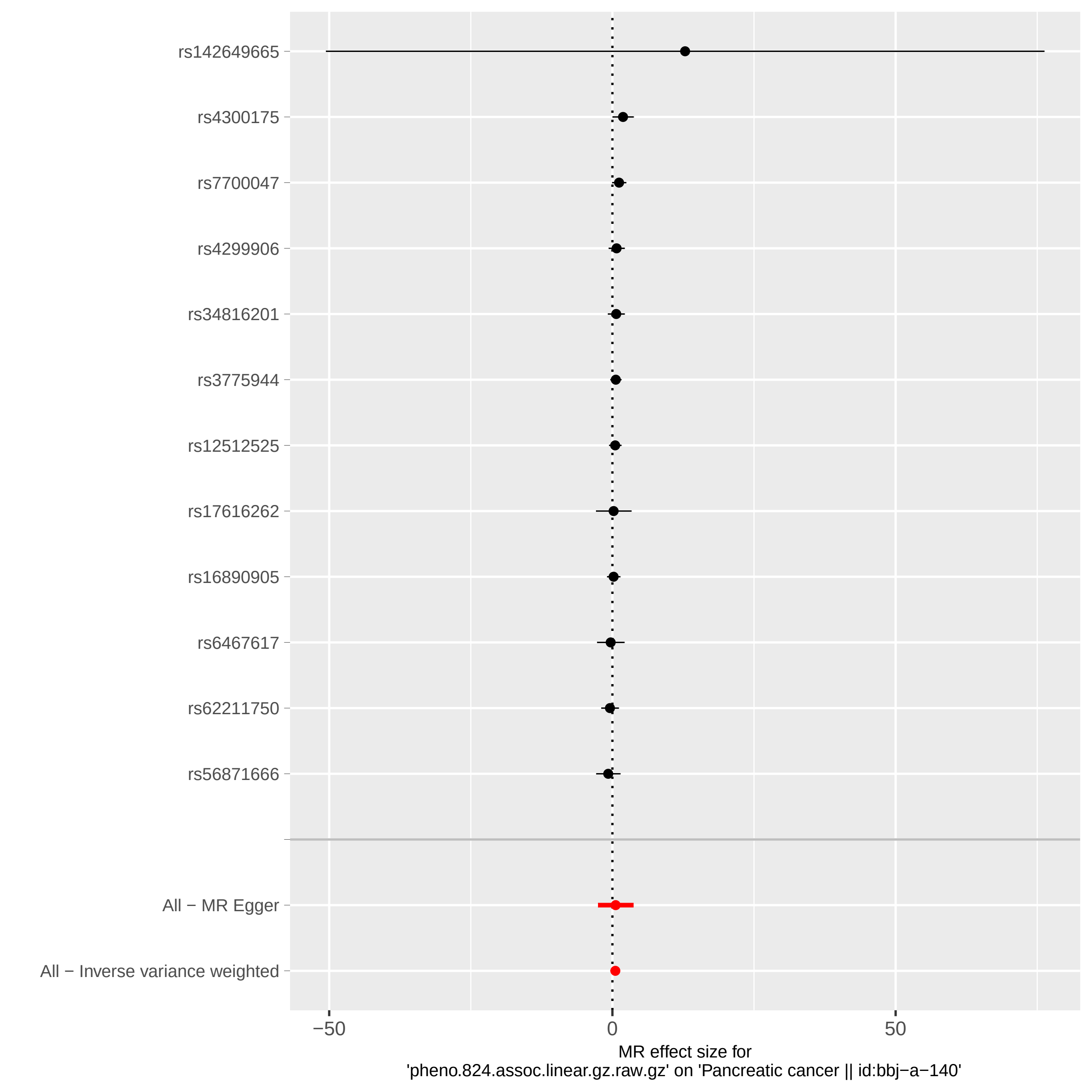

rs142649665
rs4300175
rs7700047
rs4299906
rs34816201
rs3775944
rs12512525
rs17616262
rs16890905
rs6467617
rs62211750
rs56871666
All − MR Egger
All − Inverse variance weighted
−50
0
50
MR effect size for
'pheno.824.assoc.linear.gz.raw.gz' on 'Pancreatic cancer || id:bbj−a−140'
